# The diversity of mtDNA *rns* introns among strains of *Ophiostoma piliferum*, *Ophiostoma pluriannulatum* and related species

**DOI:** 10.1186/s40064-016-3076-6

**Published:** 2016-08-24

**Authors:** Iman M. Bilto, Georg Hausner

**Affiliations:** Department of Microbiology, University of Manitoba, Winnipeg, MB R3T 2N2 Canada

**Keywords:** Group I and group II introns, Homing endonucleases, *Ophiostoma*, mtDNA

## Abstract

**Background:**

Based on previous studies, it was suspected that the mitochondrial *rns* gene within the Ophiostomatales is rich in introns. This study focused on a collection of strains representing *Ophiostoma piliferum*, *Ophiostoma pluriannulatum* and related species that cause blue-stain; these fungi colonize the sapwood of trees and impart a dark stain. This reduces the value of the lumber. The goal was to examine the mtDNA *rns* intron landscape for these important blue stain fungi in order to facilitate future annotation of mitochondrial genomes (mtDNA) and to potentially identify mtDNA introns that can encode homing endonucleases which may have applications in biotechnology.

**Results:**

Comparative sequence analysis identified five intron insertion sites among the ophiostomatoid fungi examined. Positions mS379 and mS952 harbor group II introns, the mS379 intron encodes a reverse transcriptase, and the mS952 intron encodes a potential homing endonuclease. Positions mS569, mS1224, and mS1247 have group I introns inserted and these encode intact or eroded homing endonuclease open reading frames (ORF). Phylogenetic analysis of the intron ORFs showed that they can be found in the same insertion site in closely and distantly related species.

**Conclusions:**

Based on the molecular markers examined (rDNA internal transcribed spacers and *rns* introns), strains representing *O. pilifera*, *O. pluriannulatum* and *Ophiostoma novae*-*zelandiae* could not be resolved. Phylogenetic studies suggest that introns are gained and lost and that horizontal transfer could explain the presence of related intron in distantly related fungi. With regard to the mS379 group II intron, this study shows that mitochondrial group II introns and their reverse transcriptases may also follow the life cycle previously proposed for group I introns and their homing endonucleases. This consists of intron invasion, decay of intron ORF, loss of intron, and possible reinvasion.

**Electronic supplementary material:**

The online version of this article (doi:10.1186/s40064-016-3076-6) contains supplementary material, which is available to authorized users.

## Background

Fungal mitochondrial genomes (mtDNAs) usually encode genes for the ribosomal small and large subunit RNAs (*rns*, *rnl*), tRNA, proteins involved in the respiratory chain (*cox1, cox2, cox3*, and *cob*), subunits of the NADH dehydrogenase (*nad1* to *nad6* and *nad4L*; except for members of the Taphrinomycota and some members of the Saccharomycetales), components of the ATP synthase (*atp6, atp8*, and *atp9*), and in some instances ribosomal proteins (*rps3*) (Bullerwell et al. 2003; Bullerwell and Lang 2005; Procházka et al. 2010; Solieri 2010; Eldarove et al. 2011; Freel et al. 2015). On a few occasions, mtDNA mutations can trigger senescence and in some fungal plant pathogens, hypovirulence (Bertrand 2000). With regard to metazoans, mtDNA mutations can cause degenerative mitochondrial diseases in humans (Wallace 2010). Fungal mtDNAs are highly variable both in size and organization due to various recombination events and the presence of intergenic spacers, introns, and intron-encoded open reading frames (ORFs) (Palmer et al. 2000; Mardanov et al. 2014, Aguileta et al. 2014; Wu and Hao 2014; Freel et al. 2015).

Fungal mtDNA introns tend to be self-splicing elements that can catalyze their own excision from transcripts and depending on the excision mechanism, they have been divided into group I and group II introns (Saldanha et al. 1993; Lambowitz et al. 1999). Splicing of groups I (GI) and II (GII) introns tend to be facilitated by a combination of intron-encoded (maturases) or host genome-encoded factors (Lang et al. 2007; Hausner 2012). Some of these introns have the potential to be mobile due to the presence of intron-encoded proteins (IEPs) that promote the movement of their host introns from intron-containing alleles to cognate alleles that lack the intron (Dujon 1989). Homing endonucleases (HEs) are DNA-cutting enzymes encoded by homing endonuclease genes (HEGs) and these are frequently encountered as ORFs within GI introns and in some instances within GII introns (Toor and Zimmerly 2002; reviewed in Hafez and Hausner 2012). HEGs can also be freestanding, encoded within archaeal introns, and comprise the DNA-cutting component of inteins (Gimble 2000; Belfort et al. 2002; Stoddard 2005; Barzel et al. 2011). Currently, at least six families of HEs are recognized. Their naming is based on conserved amino acid motifs: the LAGLIDADG, H–N–H, His-Cys box, PD-(D/E)xK, EDxHD, and GIY-YIG families of HEs (Stoddard 2011, 2014).

Group I and II introns are highly variable in their primary structure but both show conservation in their secondary structures. For GI introns, about 10 helical regions have been noted (P1–P10) that stabilize the intron core in folding into a splicing-competent structure (reviewed in Hausner et al. 2014). Group I introns can be assigned into various subgroups based on features related to secondary or tertiary structures and sequence peculiarities (Michel and Westhof 1990). Group II introns tend to form secondary structures that consist of six double-helical domains (domains I–VI) radiating from a central wheel. Domain V is the most conserved component with regard to the primary sequence (reviewed in Toor et al. 2001). Group II introns are assigned into several different classes based on structural features and the type of interactions between intron and exon sequences (Lambowitz and Belfort 2015). Group II introns are retroelements that encode proteins with reverse transcriptase activity (RT). Usually, GII intron mobility is promoted by a ribonucleoprotein consisting of the IEP and the spliced lariat version of the intron RNA (Lambowitz and Zimmerly 2011). With regard to GI intron-encoded proteins, there are two families of HEs that are commonly encountered within fungal mtDNAs. These are the LAGLIDADG (LHE) and GIY-YIG families of HEs (Stoddard 2005, 2011). It is worth noting that for some LAGLIDADG type ORFs, it has been shown that they can function as maturases or in some cases have two activities, promote splicing and mobility of their host intron (Szczepanek and Lazowska 1996; Bolduc et al. 2003).

Some *Ophiostoma* species are blue stain fungi [e.g., *Ophiostoma piliferum* (Fr.) Syd. & P. Syd.] and some are plant pathogens [e.g., *Ophiostoma ulmi* (Buisman) Melin & Nannf. that causes Dutch elm disease (Wingfield et al. 1993)]. Blue stain fungi cause discoloration of wood and this reduces the economic value of the lumber. Currently, very little is known about the mtDNAs for species of *Ophiostoma* and the contribution of so-called mobile introns toward mtDNA stability and diversity. For *Cryphonectria parasitica* [(Murrill) M.E. Barr], there is evidence that an *rns* GII intron could be associated with inducing hypovirulence (Baidyaroy et al. 2011). Thus, mapping and characterizing introns may have applications with regard to attenuating virulence for pathogenic members of the genus *Ophiostoma*. Furthermore, ribozymes and HEs have been shown to have applications in biotechnology (Sullenger and Gilboa 2002; Hafez and Hausner 2012, 2015). Uncovering more HEGs and autocatalytic introns adds to the reservoir of elements that can be developed into RNA trans-cleaving agents, genome editing tools, agents for targeted mutagenesis, etc. (reviewed in Hafez and Hausner 2012; Hausner et al. 2014; Stoddard 2014; Guha and Hausner 2016).

Previously, Hafez et al. (2013) surveyed the NCBI database along with sequences from species of *Ophiostoma* to assemble an *rns* intron landscape in order to identify positions that have been invaded by introns. The current study is an expansion to the aforementioned study by examining additional members of the genus *Ophiostoma* with a focus on *O. piliferum*, *O. pluriannulatum*, and related species.

## Methods

### Maintenance of fungi and DNA extraction

Fungal strains examined in this study are listed in Table 1. All reagents, unless noted were obtained from ThermoFisher Scientific Canada. Fungi were grown on malt extract agar (MEA; per liter: 1 g of yeast extract, 30 g of malt extract, and 20 g of agar) plates. In order to generate biomass for DNA extraction, fungi were grown in peptone yeast glucose broth (PYG; per liter: 1 g of peptone, 1 g of yeast extract, and 3 g of d-glucose). An Erlenmeyer flask containing fifty ml of PYG was inoculated with agar blocks (~1 × 1 mm) derived from an agar plate culture and the liquid cultures were incubated in the dark at 20 °C for 4–6 days. The protocol used for DNA extraction was previously described by Hausner et al. (1992). Briefly, fungal mycelia were harvested by vacuum filtration using Whatman filter paper #1 and collected in 15 ml centrifuge tubes. Six ml of extraction buffer [100 mM Tris–HCl (pH 8.0), 20 mM Na_2_EDTA·2H_2_O (pH 8.0), 1.5 M NaCl, 2 % (w/v) cetyltrimethylammonium bromide (CTAB)] was added along with 3 g of glass beads and 660 μl of 20 % (w/v) sodium dodecyl sulfate (SDS). This mixture was vortexed for about 2 min. Thereafter, the lysate was incubated at 55 °C for 2 h. Cell debris and contaminants were removed by chloroform extraction and centrifuging for 20 min at 2000 rpm. The top aqueous layer was recovered and 2.5 volume of ice cold 95 % ethanol was added to precipitate the nucleic acids. Finally, the nucleic acids were recovered by centrifuging for 30 min at 3000 rpm. The DNA pellets were washed with 1 ml of 70 % ethanol and resuspended in 300 µl TE buffer [10 mM Tris–HCl (pH 7.6) and 1 mM Na_2_EDTA·2H_2_O (pH 8.0)].

**Table 1:** Strains used in this study

	Species	Strain number	*rns* length (kb)
1	*Ophiostoma pluriannulatum*	WIN(M)1530 (=NZPS 1555)	1.2
2	*Ophiostoma pluriannulatum*	WIN(M)1531 (=NZPS 1552)	1.2
3	*Ophiostoma pluriannulatum*	WIN(M)1529 (=NZPS 1553)	1.2
4	*Ophiostoma pluriannulatum*	WIN(M)1572 (=DAOM 175,754)	1.2
5	*Ophiostoma perfectum*	WIN(M)823 (=CBS 636.66)	7
6	*Ophiostoma piliferum*	WIN(M)1543	1.2
7	*Ophiostoma californicum*	WIN(M)505	4.6
8	*Ophiostoma carpenteri*	WIN(M)853	5
9	*Ophiostoma carpenteri*	WIN(M)855 (=UAMH 9695)	5
10	*Ophiostoma subannulatum*	WIN(M)539	4.6
11	*Ophiostoma novae*-*zelandiae*	WIN(M)869 (=UAMH 9559)	1.2
12	*Ophiostoma pluriannulatum*	WIN(M)1561	1.2
13	*Ophiostoma pluriannulatum*	WIN(M)455 (=ATCC 8714)	1.2
14	*Pesotum* sp.	WIN(M)163	1.2
15	*Ophiostoma piliferum*	WIN(M)1548	1.2
16	*Ophiostoma novae*-*zelandiae*	WIN(M)864 (=UAHM 9557)	1.2
17	*Ophiostoma pluriannulatum*	WIN(M)1549	1.2
18	*Ophiostoma pluriannulatum*	WIN(M)1552	1.2
19	*Ophiostoma novae*-*zelandiae*	WIN(M)863 (=UAMH 9556)	1.2
20	*Ophiostoma piliferum*	WIN(M)971	1.2
21	*Ophiostoma piliferum*	WIN(M)972	4.6
22	*Ophiostoma piliferum*	WIN(M)973	1.2
23	*Ophiostoma piliferum*	UAHM 7459	1.2
24	*Ophiostoma piliferum*	UAMH 7233	1.2

*ATCC,* American Type Culture Collection, P.O. Box 1549, Manassas, VA 20108, USA; *CBS*, Centraal Bureau voor Schimmelcultures, Utrecht, The Netherlands; DAOM, Cereal and Oilseeds Research, Agriculture & Agri-Food Canada, Ottawa, Ont., Canada; *NZPS*, from Colette Breuil (University of British Columbia) collected by Roberta Farrell, University of Waikato, New Zealand; *UAMH* University of Alberta Microfungus Collection & Herbarium, Devonian Botanic Garden, Edmonton, AB, Canada, T6G 2E1; *WIN(M)*, University of Manitoba (Winnipeg) Collection

### PCR amplification of the ITS region and *rns* gene and DNA sequencing

Primers and conditions for the polymerase chain reaction (PCR) for obtaining the internal transcribed spacer (ITS) regions and mtDNA *rns* sequences were previously described by Hausner and Wang (2005) and Hafez and Hausner (2011a), respectively. Briefly, the ITS (ITS1 and ITS2) regions were amplified using the following primers: SSU-Z/LSU-4 and SSU-3/LSU-2 (Hausner and Wang 2005). Primers utilized for the amplification of the mtDNA *rns* gene were previously described by Hafez and Hausner (2011a) and Hafez et al. (2013); additional primers were designed for sequencing in order to extend reads and to close contigs (Table 2). All PCR products where analyzed on 1 % agarose gels by electrophoresis in TBE buffer [89 mM Tris–borate, 10 mM EDTA (pH 8.0)]. The 1-Kb Plus DNA Ladder (Invitrogen) was included as molecular weight markers. Gels were stained with ethidium bromide (0.5 μg/ml in 1X TBE buffer) and examined under UV light. PCR products were cleaned up using the Geneaid kit (Froggo Bio, 230 Canarctic Drive, Toronto, ON, Canada) following the protocol provided by the manufacturer. PCR fragments were sequenced by cycle sequencing utilizing the BigDye^®^ Terminator v3.1 cycle sequencing kit (Applied Biosystems, 850 Lincoln Centre Drive, Foster City, CA, 94404, USA). Automated fluorescent DNA sequence analysis was performed by the Manitoba Institute of Cell Biology DNA Sequencing Facility [675 McDermot Ave., Cancer Care Manitoba (CCMB) Building].

**Table 2:** Primers used for amplifying segments of the mtDNA *rns* gene

Primer name	Sequence (5′–3′)
rns-F0	GAGTTTGGTGATGGCTCTG
rns-F1	GCTGCCAGCAGTCGCGG
rns-F2	GGATTAGAGACCCTTGTAG
rns-F3	ACACCAGTAGTGAAGTATG
rns-R0	CCACTACACGAACCGTATTTC
rns-gp2-R1	CATTAACTGGAAACAGCCGTGCAAC
rns-R2	CTACAAGGGTCTCTAATCC
rns-R3	CCGCGACTGCTGGCACG
mtsr-1	AGTGGTGTACAGGTGAG
mtsr-2	CGAGTGGTTAGTACCAATCC

### Preliminary DNA sequence analysis

Sequencing results were assembled as FASTA-formatted files and applied to the sequence assembly program CAP3 (http://doua.prabi.fr/software/cap3). The resulting contigs were used as templates for designing new primers. Once completed, the *rns* sequences were applied to the basic local alignment search tool (BLAST; http://www.ncbi.nlm.nih.gov/Blast.cgi/) to identify similar sequences. Sequences were compiled and aligned by the online multiple sequence alignment program, MAFFT (http://mafft.cbrc.jp/alignment/server/; setting: E-INS-I; Katoh and Standly 2013). The aligned sequences were further examined with the GeneDoc program (Nicholas et al. 1997) to determine intron/exon boundaries. This was accomplished by aligning sequences with introns against those that lack introns.

### Intron folding

Folding of GI and GII introns was based on previous work by Michel and Westhof (1990), Toor et al. (2001), Lambowitz and Zimmerly (2004) and Hafez and Hausner (2011a), along with input from the online program RNAweasel (http://megasun.bch.umontreal.ca/RNAweasel/). The literature was also consulted with regard to comparing introns identified in this study with introns and their putative folds, characterized in other studies (Hafez et al. 2013 and citations within). Intron folds compiled at the RNA comparative web site (http://www.rna.icmb.utexas.edu/) were also consulted. Intron sequences (along with flanking exon sequences) were applied to RNAweasel (Lang et al. 2007). This program can identify and, in many cases, classify introns. The program can also predict the intron core sequences as it relates to the secondary structure (Lang et al. 2007). The mfold online program (http://unafold.rna.albany.edu/?q=mfold; Zuker 2003) was also used to assist in folding sections of the introns. ORF Finder (NCBI; set on genetic code 4) was used to identify potential ORFs within intron sequences. The final intron RNA folds were manually drawn using CorelDRAW Graphics Suite X6 (Corel Corporation, Ottawa).

### Phylogenetic studies

 Nucleotide and amino acid alignments were analyzed with programs contained within the MEGA 6 program package (Tamura et al. 2011). For all aligned data sets, the most suitable models for phylogenetic analysis were chosen based on the “best model” option as implemented in MEGA 6. The ITS data was aligned with ClustalX (Thompson et al. 1997) and the alignment was adjusted with GeneDoc (Nicholas et al. 1997). The ITS alignment was analyzed with neighbor joining (NJ, Maximum Composite Likelihood model), parsimony (PARS), and the maximum likelihood (ML, T92+G model) methods. In all cases, the bootstrap option was implemented (1000 replicates) in order to assess node support values. The ITS data was also analyzed with Mr. Bayes (F81 model) running 5,000,000 generations and removing (burn-in command) 40 % of sampled trees to compute the majority-rule consensus tree.

Data sets for intron-encoded ORFs were enriched by extracting sequences from NCBI databases using sequences obtained from this study as queries in blastp searches. Data sets were aligned with MAFFT and manual adjustments were made, if necessary, with GeneDoc. Amino acid (aa) alignments were analyzed with three tree building methods [neighbour joining (NJ), parsimony (PARS), and maximum likelihood (ML)] and the bootstrap option was implemented (2000 replicates) in order to assess the level of support for the tree topologies generated by the respective methods. With regard to NJ analysis, the maximum composite likelihood method and its defaults were selected along with the complete deletion of gaps option. For the PARS method, the complete deletion option was also selected. In the ML method, the best model was first determined with the “best model” function as implemented in MEGA. Therefore, for ML analysis, the Whelan and Goldman plus Freq. model was selected and the complete deletion option was selected to remove segments of the alignments that contained gaps.

## Results

### Introns within the mtDNA *rns* gene

A PCR-based survey revealed polymorphism with regard to the size of the *rns* gene among the strains of *Ophiostoma* examined in this study. Based on previous reports (Hafez and Hausner 2011a, b), PCR products of 1.2 kb are representative of *rns* genes that do not have insertions and *rns* derived amplicons greater than 1.2 kb have insertions (introns). Among the 23 strains surveyed, only the following strains, based on the size of *rns*-derived PCR amplicons, appear to have insertions within the *rns* gene: *Ophiostoma californicum* WIN(M)505 [(DeVay, R.W. Davidson & W.J. Moller) Georg Hausner, J. Reid & Klassen], *Ophiostoma pluriannulatum* WIN(M)539 [(Hedgc.) Syd. & P. Syd.], *Ophiostoma carpenteri* WIN(M)855 (J. Reid & Georg Hausner), *Ophiostoma perfectum* WIN(M)823 [(R.W. Davidson) de Hoog], and *O. piliferum* WIN(M)972. The sizes of the amplicons were as follows: *O. perfectum* WIN(M)823 yielded a 7 kb PCR amplicon while *O. pluriannulatum* WIN(M)539, *O. piliferum* WIN(M)972, *O. californicum* WIN(M)505, and *O. carpenteri* WIN(M)855 yielded *rns*-derived PCR amplicons of about 5 kb (see Table 1).

**Fig. 1:**
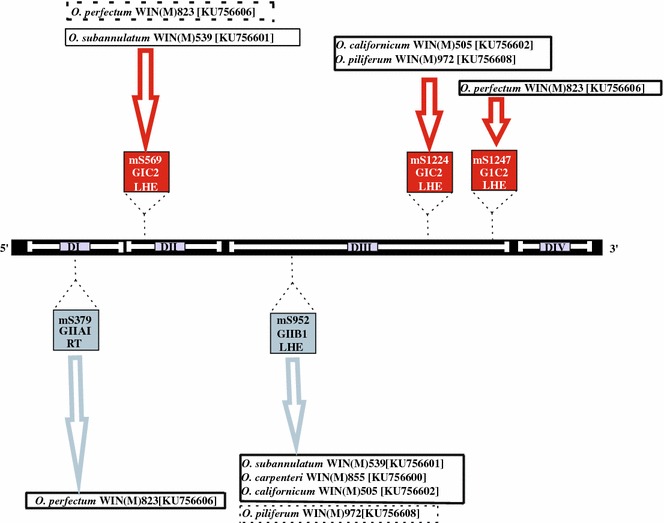
Schematic diagram depicting the mtDNA *rns* gene and its introns insertion sites for *Ophiostoma* species. The diagram shows five insertion sites and the intron classes [Group I (GI) and Group II (GII)], intron types and the intron-encoded proteins are indicated (*RT* reverse transcriptase, *LHE* LAGLIDADG type endonuclease). The *rns* structural domains are indicated by Roman numbers (I–IV) (see Hafez et al. 2013). The *red boxes* represent GI introns and the *blue boxes* represent GII introns. Names surrounded by *boxes drawn with dashed lines* indicate the presence of degraded LHEs, whereas those in *boxes with solid lines* represent introns with intact LHEs. GenBank accession numbers are also indicated

As summarized in Fig. 1, DNA sequence analysis showed that the *rns* gene of *O. perfectum* WIN(M)823 contains three introns at the following positions (with respect to the *E. coli* 16S rRNA gene; Johansen and Haugen 2001): mS379, mS569, and mS1247. The naming of introns is based on the convention of Johansen and Haugen (2001), where “m” stands for mitochondria, “S” for small ribosomal subunit gene, and the number indicates the position of the intron with reference to the *E. coli* 16S rRNA sequence. *O. piliferum* WIN(M)972 and *O. californicum* WIN(M)505 have introns inserted at positions mS952 and mS1224. *O. carpenteri* WIN(M)855 has one intron at position mS952. *O. pluriannulatum* WIN(M)539 also has an intron at mS952 in addition to an intron inserted at mS569.

### ITS analysis

**Fig. 2:**
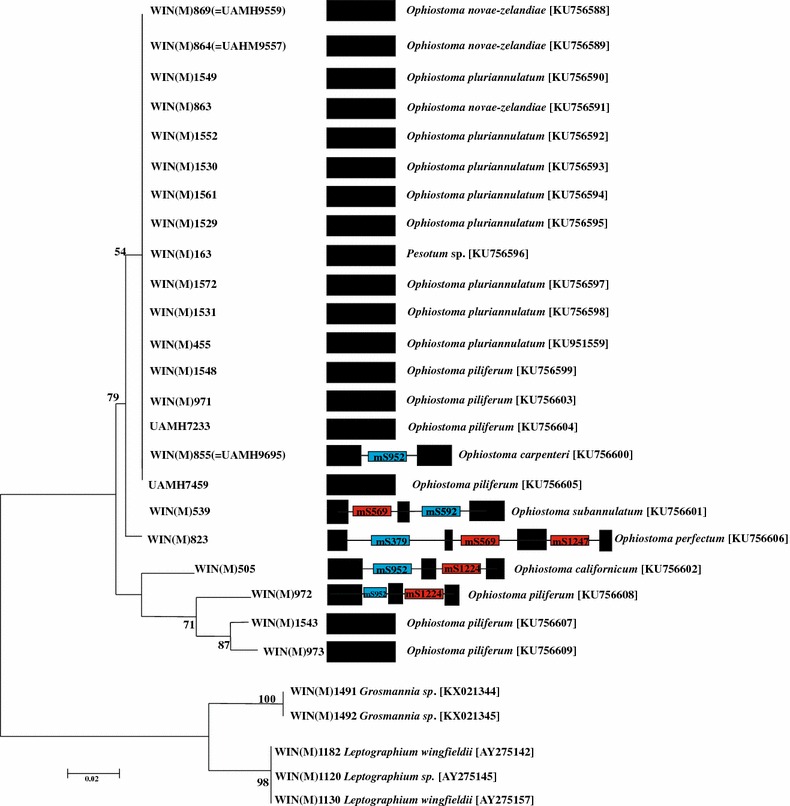
ITS based phylogeny for *Ophiostoma* species examined in this study and the schematic representation of the intron/exon combinations noted in the corresponding mtDNA rns genes. The phylogenetic tree is based on an ITS region alignment comprising 23 strains that belong to the genus *Ophistoma*. The *black boxes* represent exons and the *black lines* represent the introns. The *blue boxes* are for GII introns and the *red boxes* are for G1 introns (see Fig. 1). Tree topology is based on Mr. Bayes (MB) analysis and percentages at the nodes are node support values based in posterior probabilities (F81 model; 5,000,000 generations and burn-in of 40 %). Names of organisms and GenBank accession numbers are provided. The branch lengths are based on MB analysis and are proportional to the mean number of substitutions per site (see *scale bar*)

Phylogenetic analysis of the ITS region was used to infer relatedness of the investigated *Ophiostoma* species (Fig. 2). The ITS region was sequenced for *O. piliferum* strains along with related taxa such as *Ophiostoma novae*-*zelandiae*, *O. californicum*, *Ophiostoma subannulatum*, *O. carpenteri*, *O. perfectum*, and *O. pluriannulatum* (GenBank accession numbers: KU756588, KU756609, KX021344, KX021345). Sequences for strains representing *Leptographium* and *Grosmannia* were included as outgroups. All *Ophiostoma* species were clearly separated from the outgroup species. The taxonomy of *O. piliferum* is a complex issue (Hausner et al. 1993, 2003; Schroeder et al. 2001; De Beer et al. 2003) and it has been suggested that *O. novae*-*zelandiae* could be a synonym of *O. pluriannulatum* (Thwaites et al. 2005). The ITS phylogenetic analysis failed to accommodate the various species within their own clades (Fig. 2). In addition, although the intronless species appear to be grouped together based on the ITS sequences, the node support values for the tree do not support monophyly for this cluster.

### The *rns* introns of *Ophiostoma perfectum*

**Fig. 3:**
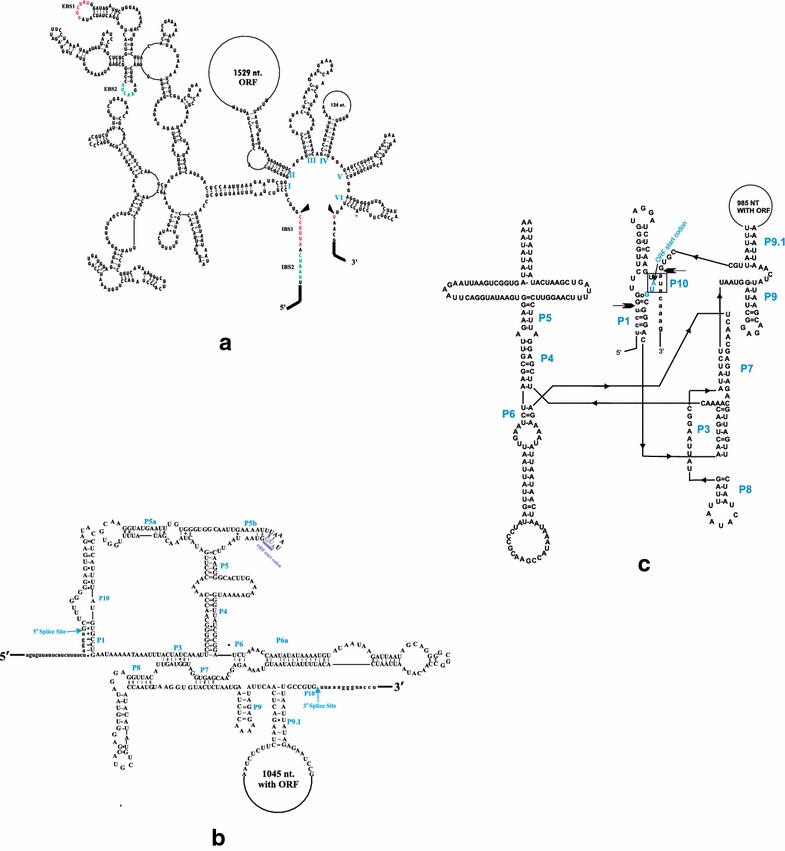
The predicted RNA folds for the *O. perfectum rns* introns. **a** Group II intron class A1 inserted at mS379; **b** a group I intron class C2 inserted at mS569; **c** group I intron class C2 inserted at mS1247. Domains (I–VI) and the exon and intron binding sequence (EBS and IBS) segments are shown for the group II introns. For the group I introns the helices (P1–P10) and conserved sequence elements are labelled. The group II intron (**a**) encodes a reverse transcriptase-type ORF within domain II. The two group I introns encode LAGLIDADG type ORFs; however, these ORFs appear to be degenerated due to the presence of premature stop codons (see text)

The *rns* gene of *O. perfectum* WIN(M)823 revealed to be the most complex with regard to intron arrangements (Fig. 2). It includes a GII intron encoding a reverse transcriptase (RT) located at position mS379 and GI introns encoding LADLIDADG type ORFs positioned at mS569 and mS1247. These introns were investigated in more detail by characterizing the potential secondary folds (Fig. 3a–c) and by evaluating the evolutionary relationships of these intron ORFs. The mS379 intron is a GII intron, type A, based on the inter domain joiners that are represented by δ and δ′ interactions, which stabilize the tertiary structure (Fig. 3a) (Lambowitz and Belfort 2015). The intron fold shows that the exon binding sites (EBS1, EBS2) within domain I are complementary to intron binding sites (IBS1, IBS2) in the upstream *rns* exon (Toor et al. 2001). This intron has an ORF embedded within domain II and the ORF encodes a RT-like protein (510 aa). The RT ORF appears to be complete with four domains that are typically associated with GII introns: RT domain, maturase domain (X), DNA binding domain (D), and an endonuclease domain (En) (Lambowitz and Zimmerly 2004).

The mS569 intron, based on structural features, is a GI C2 type (Michel and Westhof 1990) intron (Fig. 3b) as it contains P5a and P5b, and no P2. The mS569 intron contains a fragmented LHE ORF due to premature stop codons. The start codon is located within the stem of P5b and the original stop codon appears to be located within the P9.1 loop. Another GI type C2 intron has been noted at position mS1247 and it also has a degraded LHE but with a putative start codon located within the P1 stem (Fig. 3c).

### Phylogenetic analysis of *rns* intron-encoded open reading frames

The ORF Finder program identified an intact GIIA RT ORF (510 aa) in the *O. perfectum* mS379 intron. The phylogenetic tree generated for the RT data set included various fungal and bacterial group II ORFs, with *Bacillus oceanisediminis* serving as the outgroup (Additional file 1: Figure S1). The trees generated by NJ, ML and PARS were similar in topology and showed that the mS379 *O. perfectum* ORF is related to the mS379 ORF found in *Ophiostoma torulosum* [(Butin & G. Zimm.) Georg Hausner, J. Reid & Klassen] with strong bootstrap support and both of these sequences are related to the mS379 ORFs from the *Ophiostoma minus* [(Hedgc.) Syd. & P. Syd.] *rns* genes. The phylogenetic tree also shows that mS379 RT ORFs are related to an RT ORF that exists in the *cox1* gene of *Podospora anserina*. Although, at lower node support values, basal to the above grouping, are additional RT ORFs mostly encoded within *cox1* introns with the exception of the *Nitella hyalina rns* intron-encoded RT ORF. A node that received high bootstrap support (94 %) suggests monophyly for RT ORFs encoded within bacterial genomes such as *Pseudomonas syringae*, *Pseudomonas putida, Pseudomonas aeruginosa, Ktedonobacter racemifer*, and the *cox1* and *cox3* intron-encoded RT ORFs from various fungi, brown algae (*Pylaiella littoralis*) and liverwort (*Marchantia polymorpha*) along with the fungal mS379 ORFs.

The aligned data set that contained the mS569 intron ORFs, as found in both *O. perfectum* WIN(M)823 and *O. pluriannulatum* WIN(M)539, showed that the ORF is degraded in *O. perfectum* due to some frame shift mutations but otherwise nearly identical to the intact sequence found in *O*. *pluriannulatum*. A data set was assembled by extracting related sequences from GenBank depending on the *O. perfectum* WIN(M)823 sequence data as a query. The alignment was analyzed with three programs contained within MEGA6 (ML, NJ, and PARS). *Peltigera malacea* was used as an outgroup (Additional file 2: Figure S2). The *O. perfectum/O. pluriannulatum* mS569 ORFs grouped at high node support values with *rns* intron-encoded ORFs found in the following fungi: *Sclerotinia sclerotiorum*, *O. hyalothecium* and the *O. minus rns* introns. Introns that are also located at position mS569. As this phylogenetic tree overall received poor node support values, no further observations could be extracted from this analysis.

The mS952 GII intron LHE ORF from *O. carpenteri* was used to extract related sequences from GenBank. The mS952 ORF appears to be a double motif LHE in *O. carpenteri* (322 aa) and *O. pluriannulatum* WIN(M)539 (286 aa) and for *O. californicum* WIN(M)505 (323 aa) but for *O. pluriannulatum* WIN(972) (173 aa) the ORF only has one LAGLIDADG domain. The latter single domain ORF may be an indication that this ORF has eroded due to mutations that generated premature stop codons. The phylogenetic tree shows that the *O. carpenteri* GII intron-encoded LHE ORF (representing the other mS952 ORFs uncovered during this study) is found in a variety of fungi including several members of the Ophiostomatales: *Ophiostoma stenoceras* [(Robak) Melin & Nannf.], *Ophiostoma brevicolle* [(R.W. Davidson) de Hoog & R.J. Scheff.]*, O. torulosum, Leptographium* species and *O. minus*. A LHE sequence from the *nad5* i2 ORF from *Rhizoctonia solani* was used as the outgroup (Additional file 3: Figure S3). The phylogenetic analysis for the mS1224 ORFs (Additional file 4: Figure S4) recovered during this study from *O. californicum* WIN(M)505 (319aa) and *O. piliferum* WIN(M)972 (441aa) suggests that these ORFs are closely related the ms1224 intron ORFs that exists in *Ophiocordyceps sobolifera* [AB027350], *Agrocybe aegerita* [AAB50391] and *Leptosphaeria maculans* [FP929115]. The clade is supported with high node support values. The *rns* i4 intron (=mS1210) ORF from *C. parasitica* joins the above clade at a node that received only moderate support (72 %). The remaining nodes representing deeper branching patterns within this tree received only poor support values and thus, distant relationships cannot be extracted from this analysis.

Another data set was constructed by using the mS1247 intron ORF sequence from *O. perfectum* (*rns* i3 ORF) as a query in blastp. The *Amoebidium parasiticum rnl*-i2 ORF was used as the outgroup in this analysis (Additional file 5: Figure S5). The mS1247 ORF from *O. perfectum* is fragmented (three fragments) due to frameshift mutations but 418 aa could be assembled. The *O. perfectum* mS1247 ORF grouped with mS1247 ORFs from *O. sobolifera* [AB027350], *L. maculans* [FP929115] and *Chaetomium thermophilum* [JX139037]. However, the node support values are moderate for the clade which included all four mS1247 ORF sequences; but the node linking the *O. sobolifera* with *O. perfectum* received strong support (99 %). With regard to deeper nodes, the phylogenetic tree received poor node support values so no further conclusions could be reached as to possible relationships of the mS1247 with other LAGLIDADG type ORFs.

## Discussion

### *Ophiostoma* species relationships

Among the strains of *Ophiostoma* species sampled in this study, many lacked introns within the *rns* gene, but a few members did contain introns [*O. piliferum, O. carpenteri, O. pluriannulatum*, *O. perfectum* and *O. californicum*]. This is suggestive of either rapid gain or rapid loss of introns. Gain of introns could be achieved by horizontal transfer or by vertical transmission of these introns, random loss of introns could be achieved by the original intron containing allele being replaced by a cDNA version of the gene (Hausner 2012). This patterns of inheritance/maintenance of introns among the fungi have been observed among other *rns* introns (Haugen and Bhattacharya 2004; Haugen et al. 2004).

With regard to *O. piliferum*, *O. pluriannulatum* and *O. novae*-*zelandiae*, these species, based on morphological studies, tend to be easily confused with one another (Thwaites et al. 2005). Phylogenetic analysis, based on ITS sequences, also failed to distinguish among these species. Previous work, based on nuclear large ribosomal RNA subunit data, also found that currently available strains of *O. piliferum* arranged into at least two distinct clades, suggesting that *O. piliferum*, as currently circumscribed, accommodates two or more species (Hausner and Reid 2003). Although it has been proposed that the rDNA ITS region could be used as a barcode marker for the fungi (Schoch et al. 2012; Blaalid et al. 2013), this study shows that, with regard to some members of the Ophiostomatales, the ITS region may not be suitable for this purpose. Thus, more molecular markers might have to be applied to resolve these species or they may indeed be synonyms as suggested by Thwaites et al. (2005), but for the purpose of this study, the ITS data suggests we are dealing with closely related species/strains. This allows us to potentially observe more recent events with regard to the evolutionary dynamics of introns and their encoded proteins among members of the *O. piliferum* complex.

The distribution of *rns* introns also did not relate to species status, an expected result as previous studies involving Ascomycota mtDNA introns also suggested that intron-rich sequences may actually not be useful for developing DNA barcodes (Santamaria et al. 2009), i.e. the presence and absence of introns is too erratic to provide stable markers. Only a few examples exist to show that mtDNA introns could be useful in species indentification; for example the *cox1*-ai3β may have some application in identifying a subgroup within *Saccharomyces cerevisiae* (Wolters et al. 2015). The mS952 intron has been noted to be absent in strains of *Ophiostoma novo*-*ulmi* subspecies *americana*, but it has been noted to be present in strains of *Ophiosotoma ulmi* (Gibb and Hausner 2005; Hafez and Hausner 2011b).

### The *Ophiostoma rns* gene structure

This study is a continuation of the study by Hafez et al. (2013) on the intron landscape of the *rns* gene among ascomycetous fungi. However, herein we focus on the *rns* gene of *O. piliferum* and related taxa. In general, it was noted that the *rns* gene is variable in size (polymorphic) due to the presence/absence of introns and associated ORFs. Also, introns appear to be inserted at the same positions among related and distantly related fungi, which is a reflection of the homing mechanism promoted by the intron-encoded homing endonucleases. Homing endonucleases recognize long target sites and therefore, require conserved regions for their long term survival and for moving laterally from one species to another (Stoddard 2005; Belfort et al. 2002). On rare occasions, they move into new sites (ectopic integration) either by transposition or by means of reverse splicing of the intron RNA [a mechanism that has not yet been experimentally demonstrated and requires reverse transcriptase activity (Bhattacharya et al. 2005; Hausner et al. 2014)].

## The *Ophiostoma rns* introns

### The mS379 intron

The analysis of sequence data has shown that the *O. perfectum* WIN(M)832 *rns* gene is 7 kb in length and has three introns inserted in highly conserved regions within the *rns* gene. The mS379 intron is a GII intron, type AI, that was previously reported in three *O. minus* strains by Hafez and Hausner (2011a). In these three strains, the authors noted that the mS379 ORFs were at various stages of degeneration due to the presence of premature stop codons. The *O. perfectum* WIN(M)823 intron at mS379 encodes a complete RT ORF that is inserted into domain II of the GII intron. The latter is unusual as organellar GII intron ORFs are typically located in domain IV (Toor et al. 2001). Hafez et al. (2013) found related introns with a degraded ORF inserted into domain II of the mS379 GII intron in *O. torulosum* and these authors described one example of an mS379 intron that completely lacked an ORF in *O. hyalothecium*. Goddard and Burt (1999) predicted, based on their work on the yeast omega intron (mL2449/2450 GI intron in the *rnl* gene), a life cycle of invasion and degeneration for HEGs and their hosting GI introns. Our study suggests that RT ORFs in GII introns can follow a similar life cycle; i.e. invasion into an empty spot, followed by slow degeneration initially of the ORF, and eventually complete loss of the ORF, and presumably, complete loss of the intron, regenerating a potential site available for reinvasion.

A node in the mS379 tree supports monophyly for the *Ophiostoma* mS379 RT ORF within a grouping that includes sequences from brown algae, bacteria, plants and fungi. Thus, it appears this intron ORF has a complex evolutionary history that could include horizontal transfers. It has been shown that GII intron ORFs probably co-evolve with their host introns but these composite elements can evolve independently from the host genomes that encode them due to lateral transfers (Zimmerly et al. 2001; Toor et al. 2001; Toro and Nisa-Martínez 2014).

### The mS569 intron

The second intron in *O. perfectum* WIN(M)823 is the mS569 GI intron, type C2, with a LHE ORF. This intron is also found in *O. pluriannulatum* WIN(M)539. Hafez et al. (2013) found the same intron in *O. torulosum* and *O. hyalothecium*. This intron is a nice example of a phenomenon referred to as core creep (Edgell et al. 2011). Essentially, the ORF extended (“creeped”) towards the upstream exon and in some instances fused with it. This means that the ORF sequence overlaps with the intron core sequences. This might facilitate intron-encoded ORFs that are present in protein-coding genes to be more efficiently translated but among *rns* introns, it may not provide a major advantage. However, it might be a remnant of the intron’s origin, assuming it transferred from a protein-coding gene into the *rns* gene.

### The mS952 intron

In this study, four closely related species have the same intron positioned at mS952: *O. carpenteri* WIN(M)855, *O. pluriannulatum* WIN(M)927, *O. subannulatum* WIN(M)539, and *O. californicum* WIN(M)505. This GII intron was previously found in other *Ophiostoma* species by Mullineux et al. (2010, 2011), within various strains of *O. minus and O. ulmi* (Hafez and Hausner 2011a, b), and it was first noted in other fungi by Toor and Zimmerly (2002). The wide distribution of this intron suggests that it is probably, predominately, vertically inherited with some evidence of loss and possible horizontal gene transfere (HGT) (Mullineux et al. 2011). Also, the mS952 LHE ORF that is characteristic for group I introns, might be an indicator of this GII intron being potentially mobilized by a GI intron type pathway; i.e. DNA-based.

### The mS1224 intron

In a previous survey, the mS1224 intron was not seen in *Ophistoma* species but in this study, strains of *O. pluriannulatum* WIN(M)972 and *O. californicum* WIN(M)505 have a GI intron, type C2, at position S1224 and a LHE as an IEP. This intron and ORF are also found in rather distantly related fungi, *L. maculans, O. sobolifera, A. aegerita* and *C. parasitica* (Hafez et al. 2013).

### The mS1247 intron

The third intron found in *O. perfectum* WIN(M)823 is located at position mS1247. The mS1247 intron is a GI, type C2, encoding a LHE ORF. So far within *Ophiostoma*, the mS1247 intron has only been observed in *O. perfectum*. Hafez et al. (2013) had reported this intron in *O. sobolifera* and *L. maculans* (and they also noted that this site can be occupied by a twintron or nested introns, which is an intron inserted within an intron (Copertino and Hallick 1991; Michel et al. 1989; Hafez and Hausner 2015). For example, in *C. thermophilum* strain UAMH 2024, a GI intron with a double motif LHE has been invaded by an ORF-less GII intron. The latter arrangement offers a platform for designing HEs with an internal regulatory element (Guha and Hausner 2016).

## Conclusions

With regard to the *rns* intron landscape for members of the *O. piliferum* complex, this study showed that five potential intron sites exist and these are occupied by GI (S569, S1224, S1247) and GII introns (S379, S952). Among intron-encoded ORFs, the LAGLIDADG family appears to have invaded four of these introns, with the mS379 GII intron maintaining an RT ORF. Intron landscapes provide information for those that annotate mtDNAs or for those searching for sources of polymorphisms. In addition, ribozymes and intron-encoded ORFs (HEases and RTs) have applications in biotechnology, genome editing, or as functional genomics tools (Stoddard 2005; Belfort and Bonocora 2014; Enyeart et al. 2014; Hafez and Hausner 2015; Qin et al. 2016). Intron landscapes may identify LADLIDADG HEases and host introns at insertion sites that may be similar to sequences present in genes associated with pathogenicity and/or monogenic diseases. These HEs could be developed into gene targeting tools (Hafez and Hausner 2012).

Phylogenetic investigations suggest that the intron ORFs evolve rapidly and thus, most trees had poorly supported topologies. In general, among members of the Ophiostomatales, introns appear to be gained and lost frequently. The intron ORFs tree topologies do not follow the expected host species/genome trees, which hints at the possibilities of horizontal transfers of these elements among distantly related lines. Movement of introns along with their IEPs is facilitated by their encoded proteins that target specific sites in cognate intronless alleles (Colleaux et al. 1986; Dujon 1989) or by the ability of GI and GII introns to reverse splice into transcripts that need to be reverse transcribed into cDNA, followed by a recombination event that inserts the cDNA into the genome. Reverse splicing, for GI introns in particular, requires less sequence recognition and thus, it could be an efficient method for invading new sites (Roman et al. 1999; Bhattacharya et al. 2002, 2005). This work also provided some insight into the evolution of GII introns and their RT ORFs; the mS379 intron appears to follow the intron/HEG life cycle model of invasion, decay, loss and possible reinvasion as proposed by Goddard and Burt (1999).

## Additional files

10.1186/s40064-016-3076-6 A phylogenetic tree showing the relatedness of mS379 intron ORFs among group II intron-encoded reverse transcriptase amino acid sequences. Tree topology is based on ML analysis and percentages at the nodes are node support values based on bootstrap analysis (1000 replicates). Names of organisms, host genes, intron number and location/position (when available) and GenBank accession numbers are provided. The branch lengths are based on ML (Whelan and Goldman plus Freq. model) analysis and are proportional to the mean number of substitutions per site (see scale bar).
10.1186/s40064-016-3076-6 A phylogenetic tree showing the relatedness of mS569 intron-encoded ORFs among a set of GI intron-encoded LAGLIDADG ORFs. Tree topology is based on ML analysis and percentages at the nodes are node support values based on bootstrap analysis (1000 replicates). Names of organisms, host genes, intron number and location/position (when available) and GenBank accession numbers are provided. The branch lengths are based on ML (Whelan and Goldman plus Freq. model) analysis and are proportional to the mean number of substitutions per site (see scale bar).
10.1186/s40064-016-3076-6 A phylogenetic tree showing the relatedness mS952 GII intron-encoded LAGLIDADG ORFs among group I intron ecoded LAGLIDADG ORFs. Tree topology is based on ML analysis and percentages at the nodes are node support values based on bootstrap analysis (1000 replicates). Names of organisms, host genes, intron number and location/position (when available) and GenBank accession numbers are provided. The branch lengths are based on ML (Whelan and Goldman plus Freq. model) analysis and are proportional to the mean number of substitutions per site (see scale bar).
10.1186/s40064-016-3076-6 A phylogenetic tree showing the relatedness of the mS1224 intron ORFs among other GI intron-encoded LAGLIDADG ORFs. Tree topology is based on ML analysis and percentages at the nodes are node support values based on bootstrap analysis (1000 replicates). Names of organisms, host genes, intron number and location/position (when available) and GenBank accession numbers are provided. The branch lengths are based on ML (Whelan and Goldman plus Freq. model) analysis and are proportional to the mean number of substitutions per site (see scale bar).
10.1186/s40064-016-3076-6 A phylogenetic tree shwoing the relatedness of mS1247 intron ORFs among GI intron-encoded LAGLIDADG ORFs. Tree topology is based on ML analysis and percentages at the nodes are node support values based on bootstrap analysis (1000 replicates). Names of organisms, host genes, intron number and location/position (when available) and GenBank accession numbers are provided. The branch lengths are based on ML (Whelan and Goldman plus Freq. model) analysis and are proportional to the mean number of substitutions per site (see scale bar).
