# Supplementary figures and images for: The diversity of mtDNA rns introns among strains of Ophiostoma piliferum, Ophiostoma pluriannulatum and related species
Source: Springerplus. 2016 Aug 24;5(1):1408. doi: 10.1186/s40064-016-3076-6 (PMC4995192; doi:10.1186/s40064-016-3076-6)

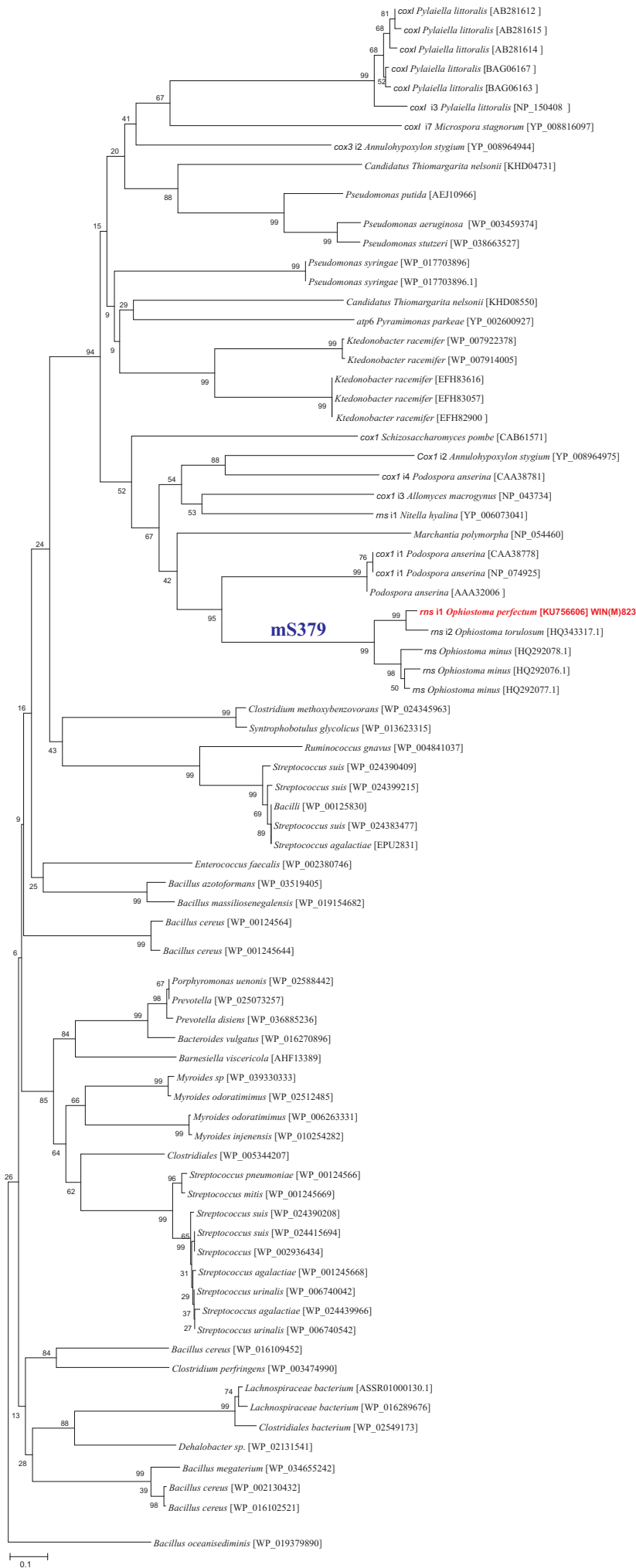

Supplement: Supplementary file 1 — 10.1186/s40064-016-3076-6 A phylogenetic tree showing the relatedness of mS379 intron ORFs among group II intron-encoded reverse transcriptase amino acid sequences. Tree topology is based on ML analysis and percentages at the nodes are node support values based on bootstrap analysis (1000 replicates). Names of organisms, host genes, intron number and location/position (when available) and GenBank accession numbers are provided. The branch lengths are based on ML (Whelan and Goldman plus Freq. model) analysis and are proportional to the mean number of substitutions per site (see scale bar). [file 40064_2016_3076_MOESM1_ESM.pdf]

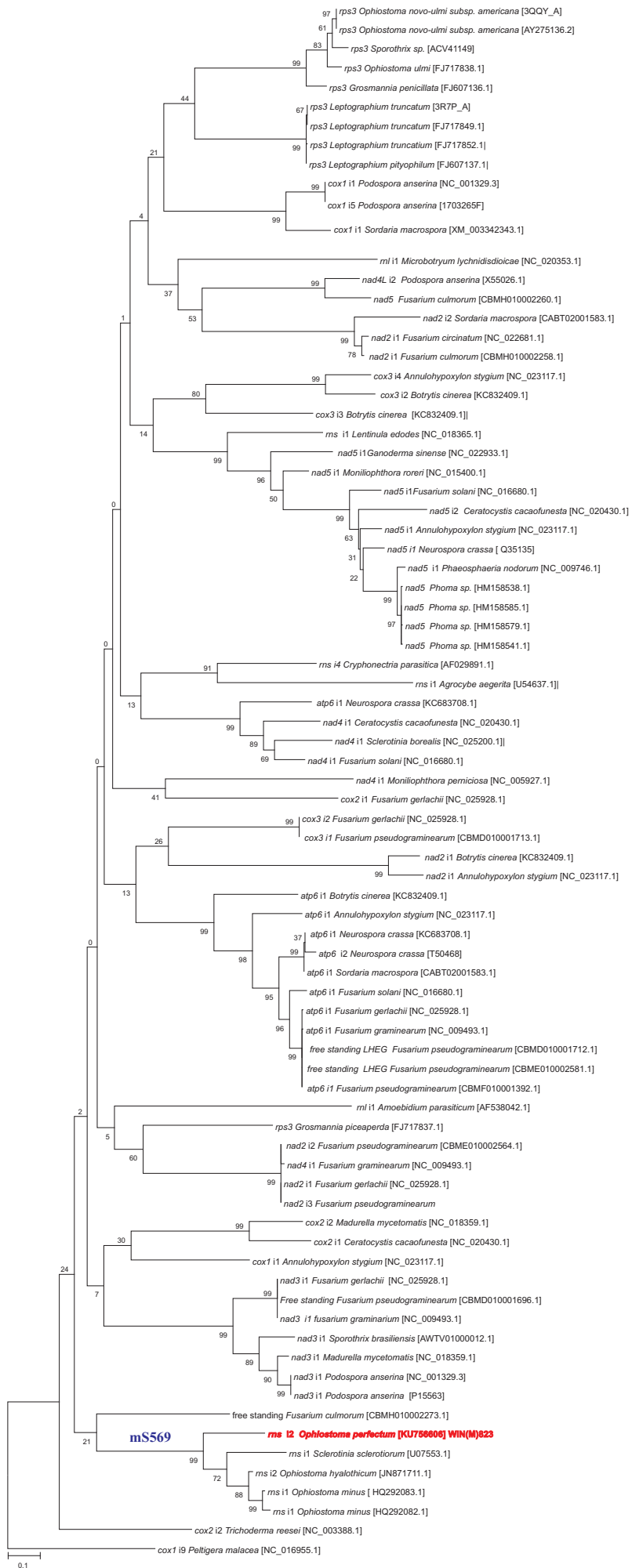

Supplement: Supplementary file 2 — 10.1186/s40064-016-3076-6 A phylogenetic tree showing the relatedness of mS569 intron-encoded ORFs among a set of GI intron-encoded LAGLIDADG ORFs. Tree topology is based on ML analysis and percentages at the nodes are node support values based on bootstrap analysis (1000 replicates). Names of organisms, host genes, intron number and location/position (when available) and GenBank accession numbers are provided. The branch lengths are based on ML (Whelan and Goldman plus Freq. model) analysis and are proportional to the mean number of substitutions per site (see scale bar). [file 40064_2016_3076_MOESM2_ESM.pdf]

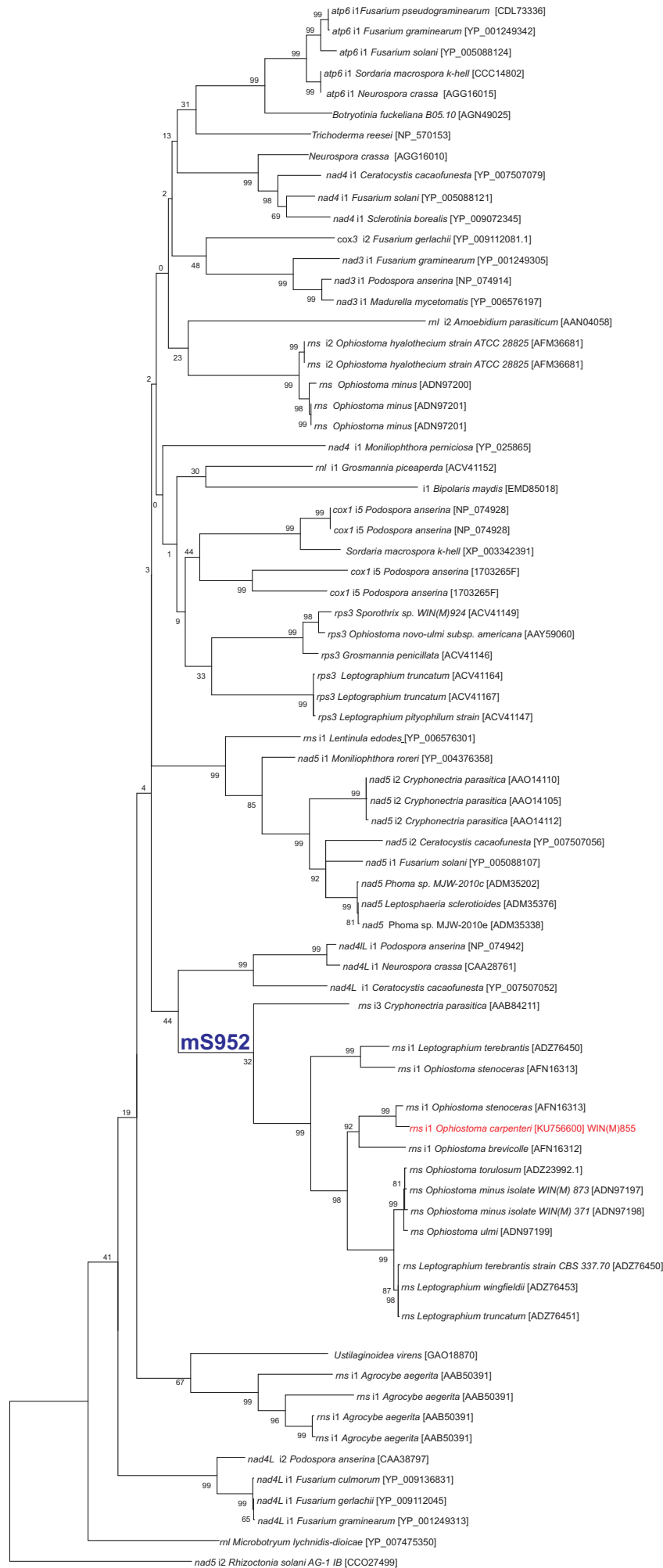

0.2

Supplement: Supplementary file 3 — 10.1186/s40064-016-3076-6 A phylogenetic tree showing the relatedness mS952 GII intron-encoded LAGLIDADG ORFs among group I intron ecoded LAGLIDADG ORFs. Tree topology is based on ML analysis and percentages at the nodes are node support values based on bootstrap analysis (1000 replicates). Names of organisms, host genes, intron number and location/position (when available) and GenBank accession numbers are provided. The branch lengths are based on ML (Whelan and Goldman plus Freq. model) analysis and are proportional to the mean number of substitutions per site (see scale bar). [file 40064_2016_3076_MOESM3_ESM.pdf]

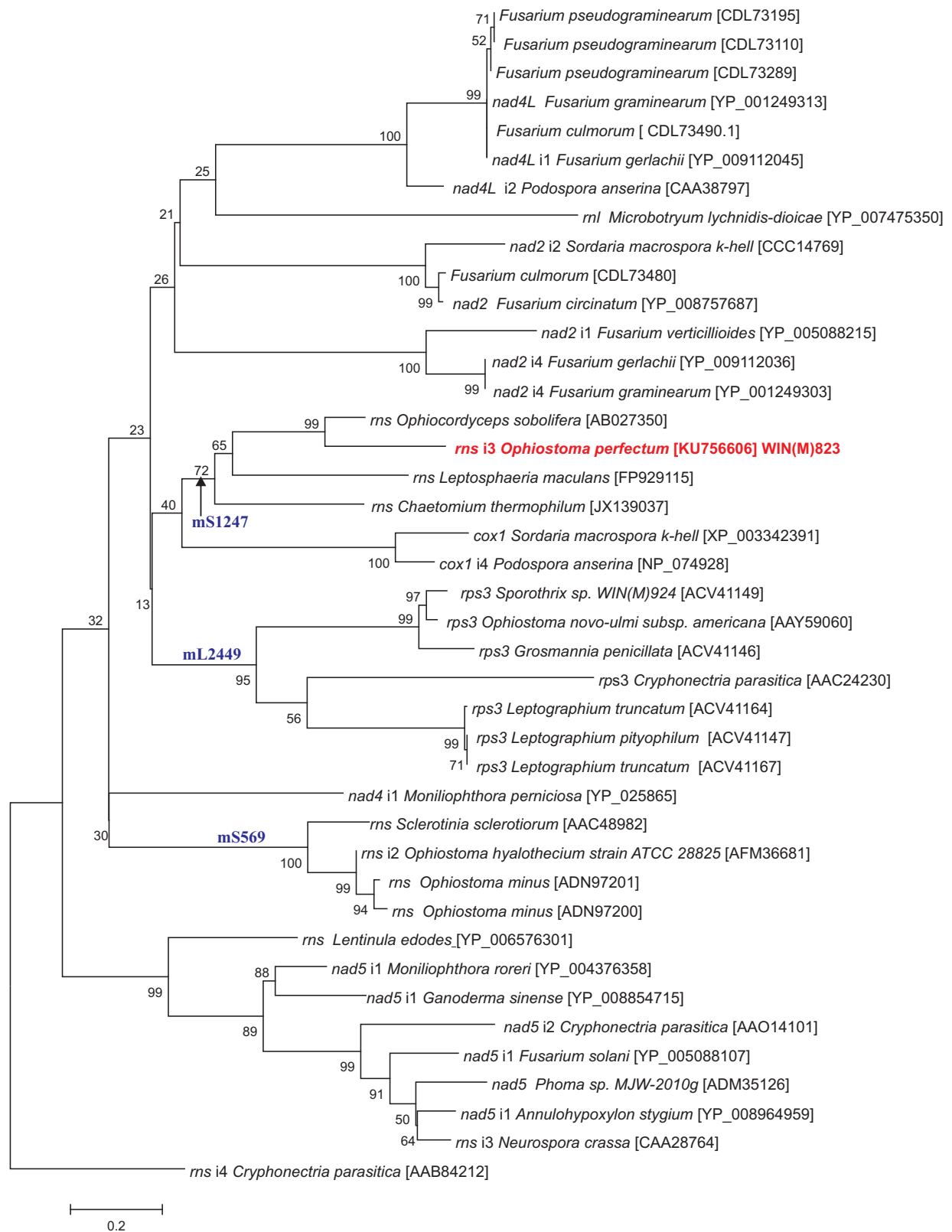

Supplement: Supplementary file 4 — 10.1186/s40064-016-3076-6 A phylogenetic tree showing the relatedness of the mS1224 intron ORFs among other GI intron-encoded LAGLIDADG ORFs. Tree topology is based on ML analysis and percentages at the nodes are node support values based on bootstrap analysis (1000 replicates). Names of organisms, host genes, intron number and location/position (when available) and GenBank accession numbers are provided. The branch lengths are based on ML (Whelan and Goldman plus Freq. model) analysis and are proportional to the mean number of substitutions per site (see scale bar). [file 40064_2016_3076_MOESM4_ESM.pdf]

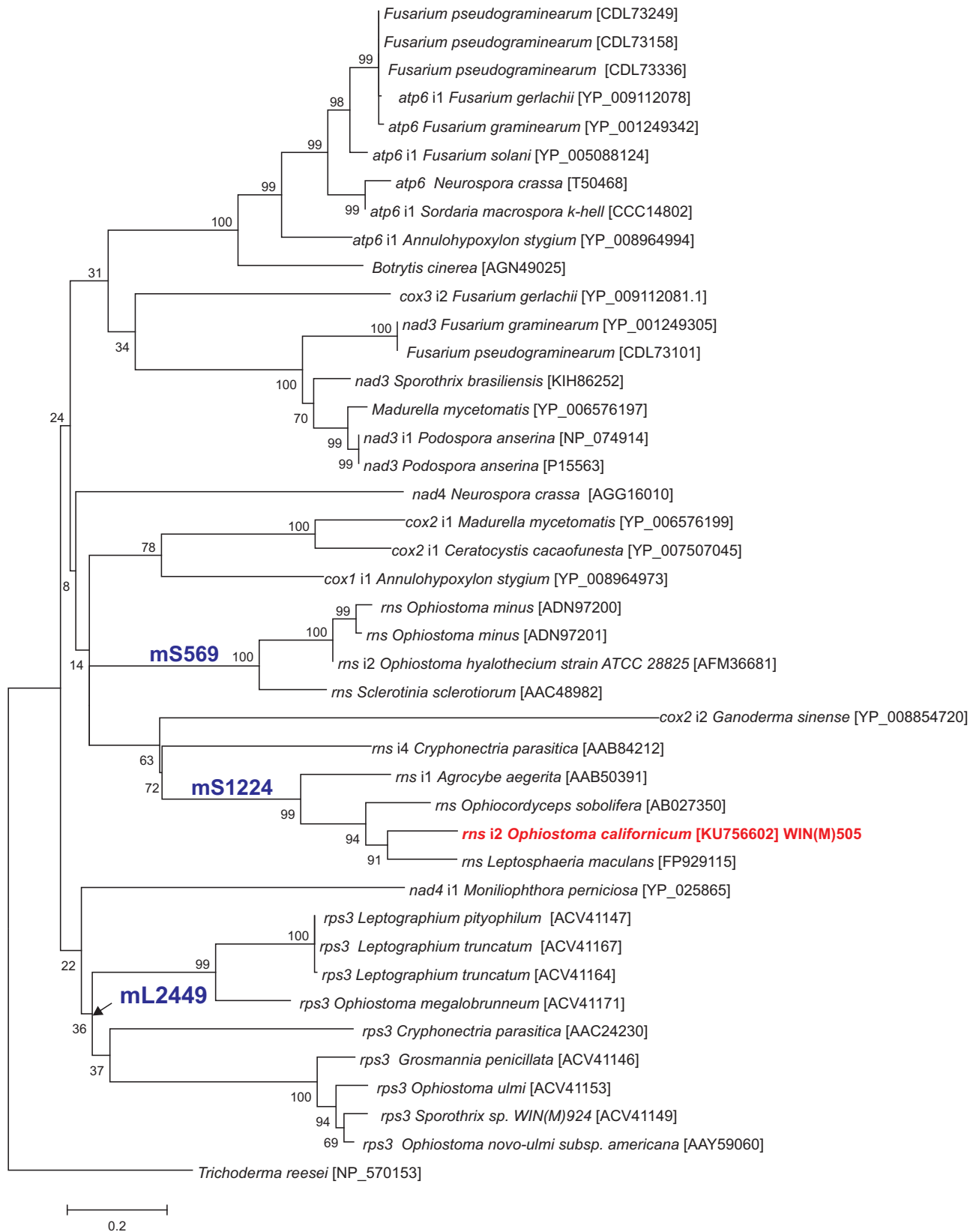

Supplement: Supplementary file 5 — 10.1186/s40064-016-3076-6 A phylogenetic tree shwoing the relatedness of mS1247 intron ORFs among GI intron-encoded LAGLIDADG ORFs. Tree topology is based on ML analysis and percentages at the nodes are node support values based on bootstrap analysis (1000 replicates). Names of organisms, host genes, intron number and location/position (when available) and GenBank accession numbers are provided. The branch lengths are based on ML (Whelan and Goldman plus Freq. model) analysis and are proportional to the mean number of substitutions per site (see scale bar). [file 40064_2016_3076_MOESM5_ESM.pdf]
